# Supplementary material for: How Enzymes, Proteins, and Antibodies Recognize Extended DNAs; General Regularities
Source: Int J Mol Sci. 2021 Jan 29;22(3):1369. doi: 10.3390/ijms22031369 (PMC7866405; doi:10.3390/ijms22031369)
Supplement: Supplementary file 1 [file ijms-22-01369-s001.pdf]

Supplementary Tables S1-S26

Supplementary Table S1

K_d values for oligonucleotides of different structure and lengths interacting with template binding site of DNA polymerase 1 (Klenow fragment)

K_d. μM*							
KH_2PO_4	26	-	-	-	-	-	-
dTMP	14.9	dAMP	13.3	dCMP	16.5	dGMP	33.0
$d(pT)_2$	9.3	$d(pA)_2$	6.8	$d(pC)_2$	7.7	$d(pG)_2$	6.5
$d(pT)_3$	4.6	$d(pA)_3$	3.4	$d(pC)_3$	4.9	-	-
$d(pT)_4$	2.6	$d(pA)_4$	1.7	$d(pC)_4$	24.0	-	-
$d(pT)_5$	1.5	$d(pA)_5$	0.85	$d(pC)_5$	2.6	-	-
$d(pT)_6$	0.82	$d(pA)_6$	0.47	$d(pC)_6$	1.6	$d(pG)_6$	0.5
$d(pT)_7$	0.46	$d(pA)_7$	0.26	$d(pC)_7$	1.1	$d(pG)_7$	0.24
$d(pT)_8$	0.26	$d(pA)_8$	0.13	$d(pC)_8$	0.69	-	-
$d(pT)_9$	0.15	$d(pA)_9$	0.065	$d(pC)_9$	0.4	-	-
$d(pT)_{10}$	0.23	$d(pA)_{10}$	0.03	$d(pC)_{10}$	0.3	$d(pG)_{10}$	0.04
$d(pT)_{11}$	0.052	$d(pA)_{11}$	0.015	$d(pC)_{11}$	0.2	-	-
$d(pT)_{12}$	0.18	$d(pA)_{12}$	0.008	$d(pC)_{12}$	0.13	-	-
$d(pT)_{15}$	0.005	$d(pA)_{15}$	0/0012	$d(pC)_{13}$	0.07	$d(pG)_{15}$	0.0014
$d(pT)_{16}$	0.0025	$d(pA)_{16}$	0.0006	$d(pC)_{15}$	0.027	-	-
$d(pT)_{17}$	0.0014	-	-	$d(pC)_{17}$	0.012	-	-
$d(pT)_{18}$	0.0009	-	-	$d(pC)_{19}$	0.0045	-	-
$d(pT)_{19}$	0.0005	-	-	$d(pC)_{21}$	0.005	-	-
$d(pT)_{20}$	0.00045	$d(pA)_{20}$	0.00008	-	-	-	-
$d(pT)_{23}$	0.0005			-	-	-	-
$d(pT)_{25}$	0.00055	$d(pA)_{25}$	0.00008	-	-		-

***The error in determining the value of K_m did not exceed 7-10%**

References:

1. Nevinskii, G.A.; Levina, A.S.; Podust, V.N.; Lavrik, O.I. Eukaryotic and prokaryotic DNA-polymerase. II. The role of internucleotide phosphate groups of a template in its binding with the enzyme. *Bioorg. Khim.* **1987,** *13,* 58-68.
2. Volchkova, V.A.; Gorn, V.V.; Kolocheva, T.I.; Lavrik, O.I; Levina, A.S. Klenow fragment of DNA-polymerase I from E. coli. III. The role of internucleotide phosphate groups of the matrix in its binding with the enzyme. *Bioorg. Khim.* **1989**, *1*5, 78-89.
3. Kolocheva, T.I.; Nevinsky, G.A.; Volchkova, V.A., Levina, A.S., Khomov, V.V., Lavrik O.I. DNA polymerase I (Klenow fragment): role of the structure and length of a template in enzyme recognition. *FEBS Lett.* **1989**, *248*, 97-100.
4. Kolocheva, T.I.; Nevinsky, G.A.; Levina, A.S.; Khomov, V.V., Lavrik, O.I. The mechanism of recognition of templates by DNA polymerases from pro- and eukaryotes as revealed by affinity modification data. *J. Biomol. Struct. Dyn.* **1991**, *9*, 169-186.

Supplementary Table S2

K_m values for primers of different structure and lengths complementary to corresponding templates in the reaction catalyzed by DNA polymerase 1 (Klenow fragment)

K_m. µM							
dTMP	45.0	dAMP	71.0	dCMP	360.0	dGMP	43.0
$d(pT)_2$	23.0	$d(pA)_2$	35.0	$d(pC)_2$	148.0	$d(pG)_2$	17.5
$d(pT)_3$	15.0	$d(pA)_3$	-	$d(pC)_3$	60.	$d(pG)_3$	7.1
$d(pT)_4$	8.0	$d(pA)_4$	12.3	$d(pC)_4$	24.0	$d(pG)_4$	2.9
$d(pT)_5$	4.2	$d(pA)_5$	-	$d(pC)_5$	10.0	$d(pG)_5$	1.2
$d(pT)_6$	2.5	$d(pA)_6$	4.3	$d(pC)_6$	-	$d(pG)_6$	-
$d(pT)_7$	1.4	$d(pA)_7$	-	$d(pC)_7$	2.0	$d(pG)_7$	0.2
$d(pT)_8$	0.65	$d(pA)_8$	1.0	$d(pC)_8$	-	$d(pG)_8$	-
$d(pT)_9$	0.45	$d(pA)_9$	0.56	$d(pC)_9$	0.35	$d(pG)_9$	-
$d(pT)_{10}$	0.23	$d(pA)_{10}$	0.31	$d(pC)_{10}$	0.14	$d(pG)_{10}$	0.013
$d(pT)_{11}$	0.16	$d(pA)_{11}$	0.35	$d(pC)_{11}$	-	$d(pG)_{11}$	-
$d(pT)_{12}$	0.18	$d(pA)_{12}$	0.18	$d(pC)_{12}$	-	$d(pG)_{12}$	-
$d(pT)_{15}$	0.1	$d(pA)_{15}$	0.06	$d(pC)_{15}$	0.58	$d(pG)_{15}$	0.022

The error in determining the value of K_m did not exceed 7-10%

References:

1. Nevinsky, G.A; Veniaminova, A.G.; Levina, A.S.; Podust, V.N.; Lavrik, O.I.; Holler E. Structure-function analysis of mononucleotides and short oligonucleotides in the priming of enzymatic DNA synthesis. *Biochemistry* **1990**, *29,* 1200-1207.
2. Kolocheva, T.I.; Nevinsky, G.A.; Levina, A.S.; Khomov, V.V., Lavrik, O.I. The mechanism of recognition of templates by DNA polymerases from pro- and eukaryotes as revealed by affinity modification data. *J. Biomol. Struct. Dyn.* **1991**, *9,* 169-186

Supplementary Table S3

K_d values for oligonucleotides of different structure and lengths interacting with human uracil DNA glycosylase

K_d, µM					
**Ligand**		**Ligand**		**Ligand**	-
dTMP	45000	dAMP	10000	dTMP	45000
$d(pT)_2$	26000	$d(pA)_2$	6500	$d(pA)_2 \times d(pT)_2$	32000
$d(pT)_3$	-	$d(pA)_3$	2000	-	-
$d(pT)_4$	870	$d(pA)_4$	-	$d(pA)_4 \times d(pT)_4$	6300
$d(pT)_5$	-	$d(pA)_5$	-	-	-
$d(pT)_6$	320	$d(pA)_6$	500	$d(pA)_6 \times d(pT)_6$	1400
$d(pT)_7$	-	$d(pA)_7$	-	-	-
$d(pT)_8$	140	$d(pA)_8$	100	$d(pA)_8 \times d(pT)_8$	320
$d(pT)_9$	-	$d(pA)_9$	-	$d(pA)_9 \times d(pT)_9$	220
$d(pT)_{10}$	25	$d(pA)_{10}$	30	$d(pA)_{10} \times d(pT)_{10}$	56
$d(pT)_{11}$	-	$d(pA)_{11}$	36	-	-
$d(pT)_{12}$	25	$d(pA)_{12}$	35	$d(pA)_{12} \times d(pT)_{12}$	60
$d(pT)_{14}$	26	$d(pA)_{15}$	35	$d(pA)_{14} \times d(pT)_{14}$	60
$d(pT)_{24}$	25	$d(pA)_{16}$	35	$d(pA)_{24} \times d(pT)_{24}$	58
-	-	-	$d(pA)_{10} \times d(pT)_4$-d(pU)-$d(pT)_4$		7.0

The error in determining the value of K_m did not exceed 7-10%

References:

1. Vinogradova, N.L.; Iamkovoi, V.I.; Tsvetkov, I.V.; Nevinskii, G.A. Interaction of uracil-DNA-glycosylase from human placenta with single-stranded deoxy- and oligoribonucleotides and their complexes. *Mol. Biol. (Mosk)* **1996**, *30*, 209-219.
2. Vinogradova, N.L.; Bulychev, N.V.; Maksakova, G.A., Johnson, F.; Nevinsky, G.A. Uracil-DNA glycosylase: interpretation of X-ray data in the light of kinetic and thermodynamic studies. *Mol. Biol. (Moscow)* **1998**, *32*, 489–499.

3. Vinogradova, N.L.; Nevinsky, G.A. Enzymes of direct, excision, and corrected systems of repair of higher and lower organisms and their biological roles. *Mol. Biol. (Mosk)* **2003**, *37*, 944-960.

Supplementary Table S4

Affinity (K_d) of bases. nucleosides. NMPs and dNMPs and other small ligands for Fpg from *Escherichia coli*.

Ligand	K_d. mM*	Ligand	K_d. mM*
NaH_2PO_4	10	-	-
Deoxyribose	157	Ribose	>133
d(pR)	8.3	pR	10
C-base	48.3	-	-
T-base	46.7	-	-
A-base	20	-	-
G-base	18.3	-	-
dTMP	8.3	UMP	6.7
dCMP	8.3	CMP	8.3
dAMP	3.3	AMP	8.7
dGMP	3.3	GMP	8.0
oxo-dGMP	0.7	-	-

***Error in K_d determination was 10-20%. average results of 3-4 measurements are given.**

References:

1. Ishchenko, A.A.; Bulychev, N.V.; Maksakova, G.A.; Johnson, F.; Nevinsky, G.A. Recognition and conversion of single- and double-stranded oligonucleotide substrates by 8-oxoguanine-DNA glycosylase from Escherichia coli. *Biochemistry (Moscow)* **1997,** *62,* 204-211.

2. Ishchenko, A.A.; Bulychev, N.V.; Maksakova, G.A.; Johnson, F.; Nevinsky, G.A. Interaction of *Escherichia coli* 8-oxoguanine-DNA glycosylase with single-stranded oligodeoxyribonucleotides and their complexes. *Mol. Biol. (Moscow)* **1998**, *32,* 454-462.

3. Ishchenko, A.A.; Koval, V.V.; Fedorova, O.S.; Douglas, K.T.; Nevinsky, G.A. Structural requirements of double and single stranded DNA substrates and inhibitors, including a photoaffinity label, of Fpg protein from *E. coli. J. Biomol. Struct. Dyn.* **1999**, *17,* 301-310.

4. Ishchenko, A.A.; Vasilenko, N.L.; Sinitsina, O.I.; Yamkovoy, V.I,; Fedorova, O.S.; Douglas, K.T/; Nevinsky, G.A. Thermodinamic, kinetic, and structural basis for recognition and repair of 8-oxoguanine in DNA by Fpg protein from *E. coli. Biochemistry* **2002**, *41,* 7540-7548.

Supplementary Table S5.
Affinities of d(pN)$_n$ deoxyribooligonucleotides (K_d) and their derivatives for Fpg from *Escherichia coli*

Ligands	K_d. mM*	Oligos	K_d. mM	K_M. mM$^{\#}$	Oligos	K_d. mM.
d(pA)$_2$	2.7	d(pT)$_2$	3.3	-	d(pC)$_2$	5.0
d(pA)$_3$	1.7	d(pT)$_3$	1.7	-	-	-
d(pA)$_4$	1.3	d(pT)$_4$	1.3	-	d(pC)$_5$	0.83
d(pA)$_6$	0.77	d(pT)$_6$	0.58	-	d(pC)$_7$	0.5
d(pA)$_8$	0.25	d(pT)$_8$		-	d(pC)$_9$	0.17
d(pA)$_{10}$	0.083	d(pT)$_{10}$	0.11	-	d(pC)$_{10}$	0.1
d(pA)$_{11}$	0.083	d(pT)$_{12}$	0.066	-	d(pC)$_{12}$	0.093
d(pA)$_{14}$	0.083	d(pT)$_{14}$	0.05	-	d(pC)$_{14}$	0.083
d(pA)$_{16}$	0.083	d(pT)$_{16}$	0.033	-	-	-
d(pA)$_{20}$	0.07	d(pT)$_{20}$	0.033	-	-	
-	-	d(pT)$_{23}$	0.030	-	-	-
d[A(pF)$_2$]$^♣$	2.7	d[T(pF)$_2$]	3.3	-	d[(pF)$_9$pT]	0.083
G11**	0.01	OG11**	0.0021	0.001	-	-
G6	0.01	OG6	0.002	0.001	-	-
C11	0.006	OG5	-	0.0012	-	-
C3	0.01	OG4	-	0.0011	-	-
C2	0.01	OG3	-	0.001	-	-
C1	0.01	OG2	0.0005	0.0013	-	-
		OG1	0.002	0.00075		

*Error in K_M. K_d values determinations was 10-20%: average results of 3-4 measurements are given; $^♣$(F) is a chemically stable analog of deoxyribose (the tetrahydrofuran derivative).
The sequences of specific 23-mer single stranded ODNs (OG oligonucleotides). where **G* is oxoG (G is simple G) and complementary to them C oligonucleotides were used:
OG1 - **G***CTCTCCCTTCCTCCTTTCCTCT; OG2 - C**G***TCTCCCTTCCTCCTTTCCTCT;
OG3 - CT**G***CTCCCTTCCTCCTTTCCTCT; OG4 - CTC**G***TCCCTTCCTCCTTTCCTCT;
OG5 - CTCT**G***CCCTTCCTCCTTTCCTCT; OG6 - CTCTC**G***CCTTCCTCCTTTCCTCT;
OG11 -CTCTCCCTTC**G***CTCCTTTCCTCT; G6 - CTCTC**G**CCTTCCTCCTTTCCTCT;
G11 - CTCTCCCTTC**G**CTCCTTTCCTCT;

References:

1. Ishchenko, A.A.; Bulychev, N.V.; Maksakova, G.A.; Johnson, F.; Nevinsky, G.A. Interaction of *Escherichia coli* 8-oxoguanine-DNA glycosylase with single-stranded oligodeoxyribonucleotides and their complexes. *Mol. Biol. (Moscow)* **1998**, *32*, 454-462.
2. Ishchenko, A.A.; Koval, V.V.; Fedorova, O.S.; Douglas, K.T.; Nevinsky, G.A. Structural requirements of double and single stranded DNA substrates and inhibitors, including a photoaffinity label, of Fpg protein from *E. coli*. *J. Biomol. Struct. Dyn.* **1999**, *17*, 301-310.
3. Ishchenko, A.A.; Vasilenko, N.L.; Sinitsina, O.I.; Yamkovoy, V.I,; Fedorova, O.S.; Douglas, K.T/; Nevinsky, G.A. Thermodinamic, kinetic, and structural basis for recognition and repair of 8-oxoguanine in DNA by Fpg protein from *E. coli*. *Biochemistry* **2002**, *41*, 7540-7548.

Supplementary Table S6.
Affinities (K_d and K_M) of mixtures and duplexes of oligonucleotides for Fpg from *Escherichia coli***

Mixture or duplex	K_I. μM***	K_M. μM♣	V_{max}. %
$d(pT)_2$+$d(pA)_2$	1966	-	-
$d(pT)_3$+$d(pA)_3$	1500	-	-
$d(pT)_4$+$d(pA)_4$	1100	-	-
$d(pT)_6$+$d(pA)_6$	267	-	-
$d(pT)_8$-$d(pA)_8$	30	-	-
$d(pT)_{10}$-$d(pA)_{10}$	6.7	-	-
$d(pT)_{12}$ –$d(pA)_{12}$	2.0	-	-
$d(pT)_{14}$-$d(pA)_{14}$	0.3	-	-
$d(pT)_{15}$-$d(pA)_{15}$	0.3	-	-
$d(pT)_{16}$-$d(pA)_{16}$	0.67	-	-
$d(pT)_{20}$-$d(pA)_{20}$	1.0	-	-
G2-C2**	0.07	-	-
G11-C11	0.067	-	-
OG1-C1	0.0067	0.06	0.04
OG2-C2	0.01	0.016	0.44
OG3-C3	-	0.015	31.0
OG5-C5	-	0.01	10.0
OG6-C6	-	0.008	100
OG11-C11	0.006	0.006	100
OG20-C20	-	0.006	0.1
OG21-C21	-	40.0	0.03

*Errors in K_M. K_d values determinations was 10-30%: average results of 3-4 measurements are given.
Oligonucleotides used.* The sequences of specific 23-mer ODNs (OG oligonucleotides). where **G is oxoG and complementaty to them C oligonucleotides were used:
OG1 - **G***CTCTCCCTTCCTCCTTTCCTCT; OG2 - C**G***TCTCCCTTCCTCCTTTCCTCT;
OG3 - CT**G***CTCCCTTCCTCCTTTCCTCT; OG4 - CTC**G***TCCCTTCCTCCTTTCCTCT;
OG5 - CTCT**G***CCCTTCCTCCTTTCCTCT; OG6 - CTCTC**G***CCTTCCTCCTTTCCTCT;
OG11 -CTCTCCCTTC**G***CTCCTTTCCTCT; OG20-CCTCTCCCTTCCTCCTTTC **G***TCT
OG21-CCTCTCCCTTCCTCCTTTCC**G***CT; G6 - CTCTC**G**CCTTCCTCCTTTCCTCT;
G11 – CTCTCCCTTC**G**CTCCTTTCCTCT.

References:
4. Ishchenko, A.A.; Bulychev, N.V.; Maksakova, G.A.; Johnson, F.; Nevinsky, G.A. Interaction of *Escherichia coli* 8-oxoguanine-DNA glycosylase with single-stranded oligodeoxyribonucleotides and their complexes. *Mol. Biol. (Moscow)* **1998**, *32*, 454-462.
5. Ishchenko, A.A.; Vasilenko, N.L.; Sinitsina, O.I.; Yamkovoy, V.I,; Fedorova, O.S.; Douglas, K.T.; Nevinsky, G.A. Thermodinamic, kinetic, and structural basis for recognition and repair of 8-oxoguanine in DNA by Fpg protein from *E. coli*. *Biochemistry* **2002**, *41*, 7540-7548.

Supplementary Table S7

Affinities (K_d and K_M) of different ligands. single-stranded oligonucleotides and their duplexes for hOGG1*

Ligand	K_d. mM	Ligand	K_d. mM	Ligand	K_d. mM
P_i	68.0×10^{-3}	dGMP	6.5×10^{-3}	oxo-dGMP	1.6×10^{-3}
dAMP	1.5×10^{-3}	dTMP	2.0×10^{-3}	dCMP	9.4×10^{-3}
$d(pA)_2$	4.4×10^{-3}	$d(pT)_2$	4.8×10^{-3}	$d(pC)_2$	9.0×10^{-3}
-	-	$d(pT)_4$	7.3×10^{-4}	$d(pC)_4$	1.5×10^{-3}
$d(pA)_6$	3.7×10^{-4}	$d(pT)_6$	3.7×10^{-4}	$d(pC)_6$	6.0×10^{-4}
$d(pA)_8$	9.0×10^{-5}	$d(pT)_8$	1.1×10^{-4}	$d(pC)_8$	1.3×10^{-4}
$d(pA)_9$	7.0×10^{-5}	$d(pT)_{10}$	$7.7\times10^{-5\dagger}$	$d(pC)_{10}$	6.7×10^{-5}
$d(pA)_{10}$	9.3×10^{-5}	$d(pT)_{11}$	5.3×10^{-5}	$d(pC)_{12}$	3.3×10^{-5}
-	-	$d(pT)_{12}$	3.0×10^{-5}	$d(pC)_{14}$	3.7×10^{-5}
$d(pA)_{14}$	3.7×10^{-5}	$d(pT)_{14}$	5.7×10^{-5}	G6**	2.4×10^{-5}
$d(pA)_{16}$	2.0×10^{-5}	$d(pT)_{16}$	4.0×10^{-5}	G11**	2.0×10^{-5}
$d(pA)_{20}$	4.3×10^{-5}	$d(pT)_{20}$	2.3×10^{-5}	OG6**	$K_M = 7.5\times10^{-8}$
$d(pA)_{23}$	2.0×10^{-5}	$d(pT)_{23}$	1.7×10^{-5}	OG11**	$K_M = 8.0\times10^{-8}$

Ligand	K_d. µM	Ligand	K_d. µM
$d(pT)_6$: $d(pA)_6$	3.9×10^{-4}	$d(pT)_{14}\times d(pA)_{14}$	1.0×10^{-5}
$d(pT)_8$: $d(pA)_8$	5.7×10^{-5}	$d(pT)_{16}\times d(pA)_{16}$	1.0×10^{-5}
$d(pT)_{10}$: $d(pA)_{10}$	$6.8\times10^{-5\dagger}$	$d(pT)_{20}\times d(pA)_{20}$	9.0×10^{-6}
$d(pT)_{12}\times d(pA)_{12}$	3.7×10^{-5}	$d(pT)_{23}\times d(pA)_{23}$	1.0×10^{-5}
OG6×C6	$K_M = 3.0\times10^{-8}$***	G6×C6	1.1×10^{-5}
OG11×C11	$K_M = 1.1\times10^{-8}$***	G11×C11	8.7×10^{-6}
-	-	cOG11×C11	1.0×10^{-6}

*Errors in K_M. K_d values determinations was 10-20%: average results of 3-4 measurements are given.

Oligonucleotides used.* The sequences of specific 23-mer ODNs (OG oligonucleotides). where **G is oxoG and complementary to them C oligonucleotides were used:

OG6 - CTCTC**G***CCTTCCTCCTTTCCTCT; OG11 -CTCTCCCTTC**G***CTCCTTTCCTCT; G6 - CTCTC**G**CCTTCCTCCTTTCCTCT; G11 – CTCTCCCTTC**G**CTCCTTTCCTCT.

References:

1. Kirpota, O.O.; Endutkin, A.V.; Ponomarenko, M.P.; Ponomarenko, P.M.; Zharkov, D.O.; Nevinsky, G.A. Thermodynamic and kinetic basis for recognition and repair of 8-oxoguanine in DNA by human 8-oxoguanine-DNA glycosylase. *Nucleic Acids Res.* **2011**, *39*, 4836–4850.

Supplementary Table S8
Affinity of APN1 for minimal ligands. their derivatives. single- and double-stranded homo-deoxy Oligonucleotides

Ligand	K_d. μM**	Ligand	K_d. μM**	Ligand	K_d. μM**
NaH_2PO_4	360	*D*-ribose	>0.17 M	d(pR)	25
dAMP	165	dTMP	163.3	dCMP	163.3
d(pF)	59.0	dGMP	166.6	-	-
Single-stranded ODNs					
$d(pA)_2$	50	$d(pT)_2$	116.6	$d(pC)_2$	140
	-	$d(pT)_3$	66.7	$d(pC)_3$	60
$d(pA)_4$	33.3	$d(pT)_4$	45	-	-
	-	-	-	$d(pC)_5$	21.7
$d(pA)_6$	17.2	$d(pT)_6$	24.6	-	-
	-	-	-	$d(pC)_7$	10
$d(pA)_8$	2.5	$d(pT)_8$	8.3	-	-
	-	-	-	$d(pC)_9$	4.7
$d(pA)_{10}$	1.66	$d(pT)_{10}$	2.5	$d(pC)_{10}$	3.33
	-	$d(pT)_{11}$	2.5	$d(pC)_{11}$	3.33
$d(pA)_{12}$	1.7	$d(pT)_{12}$	2.5	-	-
	-	-	-	$d(pC)_{13}$	3.33
$d(pA)_{14}$	1.7	$d(pT)_{14}$	2.5	-	-
	-	$d(pT)_{15}$	2.57	-	-
$d(pA)_{16}$	1.66	-	-	-	-
$d[(pF)_3pT]$**	11.7	$d(pG)_2$	102	-	-
$d[(pF)_5pT]$	5.2	$d(pG)_4$	38.3	-	-
$d[(pF)_7pT]$	2.3	$d(pG)_6$	14.4	-	-
$d[(pF)_9pT]$	1.0	$d(pG)_8$	5.4	-	-
Double-stranded ODNs					
$d(pA)_2 \cdot d(pT)_2$	36.6	$d(pA)_8 \cdot d(pT)_8$	9.3	$d(pA)_{14} \cdot d(pT)_{14}$	0.33
$d(pA)_4 \cdot d(pT)_4$	26.6	$d(pA)_{10} \cdot d(pT)_{10}$	0.33	$d(pA)_{16} \cdot d(pT)_{16}$	0.33
$d(pA)_6 \cdot d(pT)_6$	11.8	$d(pA)_{12} \cdot d(pT)_{12}$	0.36	$d(pA)_{20} \cdot d(pT)_{20}$	0.33

***Standard error in experimentally determined K_d values was 10–20%; mean of 3–4 measurements are given.**
**F is tetrahydrofuran – chemically stable analog of abasic site.

References:

1. Beloglazova, N.G.; Kirpota, O.O.; Starostin, K.V.; Ishchenko, A.A.; Yamkovoy, V.I.; Zharkov, D.O.; Douglas, K.T.; Nevinsky, G.A. Thermodynamic, kinetic and structural basis for recognition and repair of abasic sites in DNA by apurinic/apyrimidinic endonuclease from human placenta. *Nucleic Acids Res*. **2004**, *32*, 5134–5146.

Supplementary Table S9
Affinity of APN1 for specific and unspecific hetero-deoxy ODNs and their duplexes

Sequences	K_d, µM**	K_d(ss): K_d(ds)
**Nonspecific ss heterooligonucleotides**		
ss dp(CTCCCTTCCT)	3.1±0.3	-
ss dp(CTCACACACT)	2.6±0.3	-
ss dp(GAAGAGAAGA)	2.2±0.4	-
ss dp(*CTAGTCAACA*)†	2.6±0.3	-
ss d[$(pT)_7$pC$(pT)_6$]	2.5±0.3	3.0
ds d[$(pT)_7$pC$(pT)_6$]·$d(pA)_{14}$	0.83±0.1	
ss d[$(pT)_7$pG$(pT)_6$]	3.0±0.3	2.0
ds d[$(pT)_7$pG$(pT)_6$]·$d(pA)_{14}$	1.5±0.2	
ss d[$(pT)_7$pR$(pT)_6$]	0.39± 0.2	3.0
ds d[$(pT)_7$pR$(pT)_6$]·$d(pA)_{14}$	0.13±0.15	
ss d[$(pT)_7$pF$(pT)_6$]	0.5±0.1	3.0
ds d[$(pT)_7$pF$(pT)_6$]·d(pA)14	0.16±0.03	
ss d(*CTAGTCAACA*CTGTCTGTGGATAC)	2.1±0.3	4.2
ds d(*CTAGTCAACA*CTGTCTGTGGATAC)	0.5±0.1	
Specific ODN containing R abasic site ss d(*CTAGTCARCA*CTGTCTGTGGATAC)	0.35±0.3	2.7
Specific ODN containing R abasic site ds d(*CTAGTCARCA*CTGTCTGTGGATAC)	0.13±0.05	

***Standard error in experimentally determined K_d values was 10–20%; mean of 3–4 measurements are given.**

References:

Beloglazova, N.G.; Kirpota, O.O.; Starostin, K.V.; Ishchenko, A.A.; Yamkovoy, V.I.; Zharkov, D.O.; Douglas, K.T.; Nevinsky, G.A. Thermodynamic, kinetic and structural basis for recognition and repair of abasic sites in DNA by apurinic/apyrimidinic endonuclease from human placenta. *Nucleic Acids Res*. **2004**, *32*, 5134–5146.

Supplementary Table S10
Affinity of different single-stranded oligonucleotides for the first DNA-binding site of Rec A protein from *E. coli*.

Ligand	$K_{d.}$ M	Ligand	$K_{d.}$ M
PO_4^{3-}	0.5	d(pR)	0.6
dTMP	2.0×10^{-2}	dCMP	12.8×10^{-3}
$d(pT)_2$	4.0×10^{-2}	$d(pC)_2$	4.7×10^{-2}
$d(pT)_3$	7.9×10^{-3}	$d(pC)_4$	1.1×10^{-2}
$d(pT)_4$	1.0×10^{-2}	$d(pC)_6$	2.5×10^{-3}
$d(pT)_5$	5.0×10^{-3}	$d(pC)_8$	5.7×10^{-4}
$d(pT)_6$	2.5×10^{-3}	$d(pC)_{10}$	5.0×10^{-4}
$d(pT)_8$	5.0×10^{-4}	$d(pC)_{12}$	4.3×10^{-5}
$d(pT)_{10}$	2.0×10^{-4}	$d(pC)_{16}$	1.8×10^{-6}
$d(pT)_{12}$	3.5×10^{-5}	$d(pC)_{20}$	1.6×10^{-7}
$d(pT)_{14}$	8.3×10^{-6}	-	-
$d(pT)_{16}$	1.5×10^{-6}	dAMP	12.4×10^{-3}
$d(pT)_{20}$	1.0×10^{-7}	$d(pA)_2$	4.5×10^{-2}
$d(pT)_{30}$	2.3×10^{-8}	$d(pA)_4$	7.0×10^{-3}
$d(pT)_{40}$	7.0×10^{-9}	$d(pA)_6$	1.04×10^{-3}
$d(T)_8$	4.8×10^{-3}	$d(pA)_8$	5.2×10^{-4}
$d(Tp)_8$	2.5×10^{-3}	$d(pA)_{10}$	2.5×10^{-4}
$d(pTT(R)_{17}T)$	9.5×10^{-6}	$d(pA)_{12}$	1.3×10^{-4}
$[d(pT)_{10}]_{et}$	5.0×10^{-3}	$d(pA)_{14}$	8.0×10^{-5}
$d(T)_8$	4.8×10^{-3}	$d(pA)_{16}$	5.4×10^{-5}
-	-	$d(pA)_{18}$	3.2×10^{-5}
-	-	$d(pA)_{20}$	2.4×10^{-5}

[*]Standard error in experimentally determined K_d values was 10–20%; mean of 3 measurements are given.

References:

1. Bugreeva, I.P.; Bugreev, D.V.; Nevinsky, G.A.. Formation of nucleoprotein RecA filament on single-stranded DNA: Analysis by stepwise increase in ligand complexity. *FEBS J.* **2005,** 272, 2734–2745.
2. Bugreeva, I.P.; Bugreev, D.V.; Nevinskii, G.A. Physico-chemical basis of RecA nucleo-protein filament formation on single. *Mol. Biol. (Mosk)* **2005,**' *39,* 984-998.
3. Bugreeva, I.P.; Bugreev, D.V., Nevinskii, G.A. Interaction of single-stranded DNA with the second DNA-binding site of RecA nucleoprotein filament. *Mol. Biol. (Mosk)* **2007**, *41*, 524-534.

Supplementary Table S11

Maximum ATP hydrolysis values by the RecA protein in the presence of various polydeoxyribonucleotides

Polymeric DNA	Maximum ATP hydrolysis.%	Polymeric DNA	Maximum ATP hydrolysis.%
poly(dA)	1.6	poly(dG)	3.5
poly(dAT)	33.2	poly(dIT)	63.4
poly(dAC)	59.4	poly(dIX)	43.4
poly(dAG)	1.3	poly(dTX)	63.6
poly(dC)	63.1	poly(dXU)	62.1
poly(dGC)	1.5	poly(dU)	63.0
poly(dT)	61.0	poly(dI)	24.3
poly(dTG)	58.5	poly(dX)	28.4

[*]Standard error in experimentally determined *hydrolysis* was 7-10 %; mean of 3 measurements are given.

References:

Bugreeva, I.P.; Bugreev, D.V., Nevinskii, G.A. Interaction of single-stranded DNA with the second DNA-binding site of RecA nucleoprotein filament. *Mol. Biol. (Mosk)* **2007**, *41*, 524-534.

Supplementary Table S12
Affinity of different unspecific single-stranded and double-stranded oligonucleotides for *Eco*RI endonuclease

Ligand	K_d . μM	Ligand	I K_d . μM	Ligand	K_d . μM
KH_2PO_4	31000	-	-	$[(C_2H_5O)_3PO]$	740000
Deoxyribozophos phate	4600	-	-	-	-
thymine	450000	-	-	thymidine	65000
TMP	2100	AMP	2500		
$d(pT)_2$	1200	$d(pA)_2$	1000	$d(pT)_2 \times d(pA)_2$	830
$d(pT)_3$	480	-			
$d(pT)_4$	360	$d(pA)_4$	250	$d(pT)_4 \times d(pA)_4$	290
$d[(pR)_3pT]$	320	-	-	-	-
$d(pT)_6$	63	$d(pA)_6$	83	$d(pT)_6 \times d(pA)_6$	110
$d(pT)_8$	48	$d(pA)_8$	56	$d(pT)_8 \times d(pA)_8$	49
$d(pT)_{10}$	42	$d(pA)_{10}$	52	$d(pT)_{10} \times d(pA)_{10}$	23
$d[p(Et)T]_{10}$	390	-	-	-	-
$d[(pR)_9pT]$	35	-	-	-	-
$d(pT)_{11}$	40	-	-	-	-
$d(pT)_{12}$	28	$d(pA)_{12}$	29	$d(pT)_{12} \times d(pA)_{12}$	12
-	-	$d(pA)_{14}$	23	-	
$d(pT)_{15}$	23	$d(pA)_{15}$	33	$d(pT)_{15} \times d(pA)_{15}$	10
$d(pT)_{16}$	17	$d(pA)_{20}$	11	-	
d(CGG)	400	d(AATTC)	62	Specific d(CGGAATTC CGTACTAC)	0.1
d(GGA)	290	d(GAATTC)	24		
d(AGA)	240	d(CGGAATTC)	4.5	-	-
d(GAC)	420	d(CGGAATC)	10	-	-

[*]Standard error in experimentally determined K_d values was 10–20%; mean of 3 measurements are given.

References:

1. Kolocheva, T.I.; Demidov, S.A.; Maksakova, G.A,; Nevinskii, G.A. Interaction of endonuclease *EcoRI* with short specific and nonspecific oligonucleotides. *Mol, Biol, (Mosk)* **1998**, *32*,1025-1033.
2. Kolocheva, T.I.; Maksakova, G.A,; Bugreev, D,?,; Nevinsky, G,A. Interaction of endonuclease *Eco*RI with short specific and nonspecific oligonucleotides. *IUBMB Life* **2001**, *51*, 189-195.
3. Kubareva, E.A.; Volkov, E.M.; Vinogradova, N.L.; Kanevsky, I.A.; Oretskaya, T.S.; Kuznetsova, S.A.; Brevnov, M.G.; Gromova, E.S.; Nevinsky, G.A.; Shabarova, Z,A. Modified substrates as probes for studing uracil-DNA glycosylase. *Gene* **1995**, *157*, 167-171

Supplementary Table S13
Affinity of different unspecific single-stranded and double-stranded oligonucleotides for HIV-1 integrase

	K_d. µM						
**Ligand**		**Ligand**	-	**Ligand**	-	**Ligand**	**Ligand**
**T-base**	> 500 000	**C-base**	> 500 000	**A-base**	> 500 000	**d-ribose**	> 500 000
**Pi**	**33 000**	**-**	**-**	**-**	**-**	**d(pR)**	**15 000**
**dTMP**	**767**	**dCMP**	**15 000**	**dAMP**	**5 000**	**-**	**-**
**$d(pT)_2$**	**330**	**$d(pC)_2$**	**300**	**$d(pA)_2$**	**80**	**-**	**-**
**$d(pT)_3$**	**166**	**$d(pC)_3$**	**100**	**$d(pA)_3$**	**48**	**-**	**-**
**$d(pT)_4$**	**73**	**$d(pC)_4$**	**50**	**$d(pA)_4$**	**3.5**	**$d(pR)_3(T)$**	**100**
**$d(pT)_5$**	**43**	**$d(pC)_5$**	**32**	**$d(pA)_6$**	**1.2**	**-**	**-**
**$d(pT)_6$**	**33**	**-**	**-**	**$d(pA)_8$**	**1.0**	**$d(pR)_7(T)$**	**60**
**$d(pT)_7$**	**26**	**$d(pC)_7$**	**10**	**$d(pA)_{10}$**	**0.52**	**-**	**-**
**$d(pT)_{10}$**	**8.3**	**-**	**-**	**$d(pA)_{12}$**	**0.40**	**-**	**-**
**-**	**-**	**$d(pC)_{16}$**	**0.100**	**$d(pA)_{14}$**	**0.30**	**$d(pR)_{13}(T)$**	**15**
**$d(pT)_{21}$**	**1.0**	**$d(pC)_{21}$**	**0.012**	**$d(pA)_{21}$**	**0.016**	**-**	**-**

**Ligand**	**K_d. µM**	**Ratio : K_d(ss)/K_d(ds) ss: $d(T)_n$ /ds: $d(T)_n$•$d(A)_n$**	**Ratio : K_d (ss) / K_d (ds) ss: $d(A)_n$/ds : $d(T)_n$•$d(A)_n$**
**$d(pT)_3 \times d(pA)_3$**	**45**	**3.6**	**1.06**
**$d(pT)_4 \times d(pA)_4$**	**3.2**	**23**	**1.1**
**$d(pT)_6 \times d(pA)_6$**	**0.6**	**55**	**2.0**
**$d(pT)_8 \times d(pA)_8$**	**0.28**	**57**	**3.6**
**$d(pT)_{10} \times d(pA)_{10}$**	**0.15**	**55**	**3.4**
**$d(pT)_{12} \times d(pA)_{12}$**	**0.078**	**70**	**5.1**
**$d(pT)_{14} \times d(pA)_{14}$**	**0.054**	**83**	**5.6**
**$d(pT)_{21} \times d(pA)_{21}$**	**0.040**	**25**	**4.0**

***Standard error in experimentally determined K_d values was 10–20%; mean of 3 measurements are given.**

References:

1. Caumont, A.; Jamieson, G.; Richard de Soultrait, V.; Parissi, V., Fournier, M.; Zakharova, O.D.; Bayandin, R.; Litvak, S.; Tarrago-Litvak, L.; Nevinsky, G.A. High affinity interaction of HIV-1 integrase with specific and non-specific single-stranded short oligonucleotides. *FEBS Lett.* **1999**, *455*, 154-158.
2. Bugreev, D.V.; Baranova, S.; Zakharova, O.D.; Parissi, V.; Desjobert, C.; Sottofattori, E.; Balbi, A.; Litvak, S.; Tarrago-Litvak, L.; Nevinsky, G.A. Dynamic, thermodynamic, and kinetic basis for recognition and transformation of DNA by human immunodeficiency virus type 1 integrase. *Biochemistry* **2003**, ***42***, 9235-9247.

Supplementary Table S14
Affinity of different unspecific single-stranded hetero-oligonucleotides for HIV-1 integrase

ODNs (sequence)	N° of units	K_d. μM
CAT	3	20
AATT	4	140
GGAA	4	33
$(GA)_3$	6	1
$(AC)_3$	6	33
CTAGCAp	6	2.6
CpAp [Gp(Et)]T**	4	70
CpAp(Et)GpT	4	40
[Cp(Et)]ApGpT	4	300
Cp(Et)Ap(Et)Gp(Et)T	4	600
$d[Tp(Et)]_9T$	10	8000
$d[(pT_9]p(ddT)$	10	15
$d(pT_8)GT$	10	8.3
d(GAGATCGTC)rA	10	0.5
$d(pAC)(pA)_8$	10	18
CCAACTTTT	9	8.3
TCACCTCCTT	10	6.6
CTGCGTCTATCAGCG	15	0.270
TTTTCCTCTCTCCCTCT	17	0.200
GGAAAATCTCTAGCAGT	17	0.033
GTGTGGAAAATCTCTAGCAGT	21	0.010
CCCTCCTCCTTCTCTCTCCTT	21	0.013
CCCTCCTCCTTCTCTCTTTTT	21	0.100

***Standard error in experimentally determined K_d values was 10–20%; mean of 3 measurements are given.**

References:

3. Caumont, A.; Jamieson, G.; Richard de Soultrait, V.; Parissi, V., Fournier, M.; Zakharova, O.D.; Bayandin, R.; Litvak, S.; Tarrago-Litvak, L.; Nevinsky, G.A. High affinity interaction of HIV-1 integrase with specific and non-specific single-stranded short oligonucleotides. *FEBS Lett.* **1999**, *455*, 154-158.

4. Bugreev, D.V.; Baranova, S.; Zakharova, O.D.; Parissi, V.; Desjobert, C.; Sottofattori, E.; Balbi, A.; Litvak, S.; Tarrago-Litvak, L.; Nevinsky, G.A. Dynamic, thermodynamic, and kinetic basis for recognition and transformation of DNA by human immunodeficiency virus type 1 integrase. *Biochemistry* **2003**, ***42***, 9235-9247.

Supplementary Table S15
Affinity of different specific single-stranded ODNs corresponding to the 3'-end processing GT- and complementary CA-strand of HIV DNA for HIV-1 integrase

5'- GTGTGGAAAATCTCTAGCA↓**GT** -3'
3'- CACACCTTTTAGAGATCGT C A -5

3'-GT-specific ODNs	N	$K_{d.}$ μM	3'-CA-specific ODNs	N	
GT	2	130	CA	2	15
AGT	3	53.3	GCA	3	3.5
CAGT	4	23.3	AGCA	4	1
GCAGT	5	13	TAGCA	5	0.7
AGCAGT	6	6.1	CTAGCA	6	0.4
TAGCAGT	7	3.3	TCTAGCA	7	0.3
CTAGCAGT	8	2.5	CTCTAGCA	8	0.2
CTCTAGCAGT	10	0.5	ATCTCTAGCA	10	0.1
AAATCTCTAGCAGT	14	0.18			
-	-	-	GGAAAATCTCTAGCA	15	0.075
GAAAATCTCTAGCAGT	16	0.063			
GTGTGGAAAATCTCTAGCAG••	19	0.03	GTGTGGAAAATCTCTAGCA	19	0.03
GTGTGGAAAATCTCTAGCAG•	20	0.15	-	-	-
GTGTGGAAAATCTCTAGCAGT	21	0.01	-	-	-
T_{19}GT	21	0.4	-	-	-
TTTTGTAAAACCACGGCCAGT	21	0.17	-	-	-
GTGTGGAAAATCTCTAGCAGrU	21	0.083	-	-	-
Specific double stranded ODNs		$K_{d.}$ μM	Ratio : K_d ss/K_d ds	-	-
AGT•d(N)$_3$	3	40	1.33	-	-
CAGT•d(N)$_4$	4	10	2.33	-	-
AGCAGT•d(N)$_6$	6	0.18	33.9	-	-
CTAGCAGT•d(N)$_8$	8	0.032	78	-	-
CT CTAGCAGT•d(N)$_{10}$	10	0.010	50	-	-
AAATCTCTAGCAGT•d(N)$_{14}$	14	0.0032	56.3	-	-
GAAAATCTCTAGCAGT•d(N)$_{16}$	16	0.0020	31.5	-	-
GTGTGGAAAATCTCTAGCA• d(N)$_{19}$	19	0.0040	7.5	-	-
GTGTGGAAAATCTCTAGCAG• d(N)$_{20}$	20	0.06	2.5	-	-
GTGTGGAAAATCTCTAGCAGT•d(N)$_{21}$	21	0.0013	7.7	-	-
T_{19}GT•A_{19}CA	21	0.036	11.1	-	-
GTGTGGAAAATCTCTAGCAGrU•d(N)$_{21}$	21	0.05	1.66	-	-

[*]Standard error in experimentally determined K_d values was 10–20%; mean of 3 measurements are given.

References:

1. Caumont, A.; Jamieson, G.; Richard de Soultrait, V.; Parissi, V., Fournier, M.; Zakharova, O.D.; Bayandin, R.; Litvak, S.; Tarrago-Litvak, L.; Nevinsky, G.A. High affinity interaction of HIV-1 integrase with specific and non-specific single-stranded short oligonucleotides. *FEBS Lett.* **1999**, *455*, 154-158.
2. Bugreev, D.V.; Baranova, S.; Zakharova, O.D.; Parissi, V.; Desjobert, C.; Sottofattori, E.; Balbi, A.; Litvak, S.; Tarrago-Litvak, L.; Nevinsky, G.A. Dynamic, thermodynamic, and kinetic basis for recognition and transformation of DNA by human immunodeficiency virus type 1 integrase. *Biochemistry* **2003**, ***42***, 9235-9247.

Supplementary Table S16
Affinity of different ligands and unspecific single-stranded ODNs for human topoisomerase I

Ligand	K_d. M	Ligand	K_d. M	Ligand	K_d. M
P_i	0.38	d(pT)$_2$	3.4×10^{-5}	d(pA)$_2$	5.2×10^{-5}
		d(pT)$_3$	3.7×10^{-3}	d(pA)$_3$	2.1×10^{-5}
d(pC)$_2$	8.2×10^{-4}	d(pT)$_4$	7.9×10^{-3}	d(pA)$_4$	1.2×10^{-3}
d(pC)$_4$	3.8×10^{-5}	d(pT)$_5$	2.6×10^{-3}	d(pA)$_6$	6.1×10^{-5}
d(pC)$_6$	6.7×10^{-5}	d(pT)$_6$	1.2×10^{-3}	d(pA)$_8$	3.3×10^{-4}
d(pC)$_8$	2.3×10^{-4}	d(pT)$_8$	1.7×10^{-3}	d(pA)$_{10}$	2.3×10^{-3}
d(pC)$_{10}$	7.3×10^{-4}	d(pT)$_{10}$	5.0×10^{-4}	d(pA)$_{12}$	7.5×10^{-4}
d(pC)$_{12}$	5.8×10^{-5}	d(pT)$_{12}$	1.3×10^{-4}	d(pA)$_{14}$	1.1×10^{-4}
d(pC)$_{14}$	1.0×10^{-5}	d(pT)$_{14}$	4.0×10^{-5}	d(pA)$_{16}$	8.3×10^{-6}
d(pC)$_{16}$	2.1×10^{-6}	d(pT)$_{16}$	1.1×10^{-5}		

***Standard error in experimentally determined K_d values was 10–15%; mean of 3 measurements are given.**

References:

1. Bugreev, D.V.; Buneva, V.N.; Sinitsina, O.I.; Nevinsky, G.A. Mechanism of recognition of supercoiled DNA by eukaryotic DNA topoisomerases I: Interactions of enzymes with nonspecific oligonucleotides". *Bioorg. Khim. (Moscow)* **2003**. *29*, 163–174.
2. Bugreev, D.V.; Buneva, V.N.; Sinitsina, O.I.; Nevinskii, G.A. The mechanism of supercoiled DNA recognition by eukaryotic type I topoisomerases. II. A comparison of the enzyme interaction with specific and nonspecific oligonucleotides", *Bioorg. Khim. (Moscow)* **2003**, ***29,*** 277-289.
3. Bugreev, D.V.; Buneva, V.N.; Nevinsky, G.A. Mechanism of supercoiled DNA cleavage by human DNA topoisomerase I: Effect of ligand structure on the catalytic stage of the reaction", *Mol. Biol. (Moscow)* **2003**. *37*, 325–339.

Supplementary Table S17
Affinity of different single-stranded ODNs corresponding to different parts of a specific DNA sequence (AAGACTTAGT) for human topoisomerase

Ligand	K_d. M	Ligand	K_d. M	Ligand	K_d. M
pAA	5.2×10^{-5}	AAGACTTAGT	$1.6\bullet10^{-6}$	AAGACTTAGT	1.6×10^{-6}
pTT	3.4×10^{-5}	TAG	4.0×10^{-4}	ACTT	1.0×10^{-4}
pTA	2.6×10^{-3}	CTT	1.0×10^{-3}	pACTT	2.0×10^{-6}
pAT	2.4×10^{-5}	pCTT	1.5×10^{-4}	GACT	2.0×10^{-4}
pCT	9.4×10^{-5}	ACT	8.0×10^{-4}	pGACT	2.1×10^{-4}
pTC	2.1×10^{-3}	GAC	1.0×10^{-4}	AGAC	3.0×10^{-5}
pCC	8.2×10^{-4}	AGA	1.5×10^{-5}	pAGAC	3.2×10^{-5}
pGG	1.0×10^{-4}	AAG	1.5×10^{-4}	AAGA	1.0×10^{-5}
pGA	9.0×10^{-5}	-	-	-	-
pAG	6.0×10^{-5}	-	-	-	-
TpT	3.0×10^{-4}	-	-	-	-
TpTp	5.6×10^{-5}	-	-	-	-
ApA	6.4×10^{-4}	-	-	-	-

***Standard error in experimentally determined K_d values was 10–15%; mean of 2-3 measurements are given.**

References:

1. Bugreev, D.V.; Buneva, V.N.; Sinitsina, O.I.; Nevinsky, G.A. Mechanism of recognition of supercoiled DNA by eukaryotic DNA topoisomerases I: Interactions of enzymes with nonspecific oligonucleotides". *Bioorg. Khim. (Moscow)* **2003**. *29*, 163–174.
2. Bugreev, D.V.; Buneva, V.N.; Sinitsina, O.I.; Nevinskii, G.A. The mechanism of supercoiled DNA recognition by eukaryotic type I topoisomerases. II. A comparison of the enzyme interaction with specific and nonspecific oligonucleotides", *Bioorg. Khim. (Moscow)* **2003**, ***29,*** 277-289.
3. Bugreev, D.V.; Buneva, V.N.; Nevinsky, G.A. Mechanism of supercoiled DNA cleavage by human DNA topoisomerase I: Effect of ligand structure on the catalytic stage of the reaction", *Mol. Biol. (Moscow)* **2003**. *37*, 325–339.

Supplementary Table S18
Affinity of different single- and double-stranded ODNs corresponding to different parts of a specific DNA sequence for human topoisomerase before and after reaction mixture preincubation

Ligand	K_d (1), μM (before preincubation)	K_d (2), μM (after preincubation)	Ratio: K_d (1)/K_d (2)
AAGTC (NC5)	92.0	8.3	11.0
GACTT (C5)	2.0	0.40	5.0
CTAAGTCTT (NC9)	2.1	0.17	12.3
AAGACTTAG (C9)	1.6	0.32	5.0
NC27**	166.0	8.3	20.0
C27**	83.0	4.2	19.8
C5+NC5	7.5	1.0	7.5
C9+NC5	0.65	0.16	4.0
C9+C5	25.0	0.6	42.0
C5+NC9	29.0	2.0	14.5
C9+NC9	0.65	0.085	7.6
NC27+C27	0.07	0.01	7.0
AAGAAATAG	2.2	-	-
AAGACTTAGp	1.6	-	-
AAGACTT	20.0	-	-
AAGACTTp	1.1	-	-

***Standard error in experimentally determined K_d values was 10–20%; mean of 3 measurements are given.**
**C27. AAAAAGACTTAGAAAAATTTTTAAAG;
NC27.CTTTAAAAATTTTTCTAAGTCTTTTTT.

References:

1. Bugreev, D.V.; Buneva, V.N.; Sinitsina, O.I.; Nevinsky, G.A. Mechanism of recognition of supercoiled DNA by eukaryotic DNA topoisomerases I: Interactions of enzymes with nonspecific oligonucleotides”. *Bioorg. Khim. (Moscow)* **2003**. *29,* 163–174.
2. Bugreev, D.V.; Buneva, V.N.; Sinitsina, O.I.; Nevinskii, G.A. The mechanism of supercoiled DNA recognition by eukaryotic type I topoisomerases. II. A comparison of the enzyme interaction with specific and nonspecific oligonucleotides”, *Bioorg. Khim. (Moscow)* **2003**, ***29,*** 277-289.
3. Bugreev, D.V.; Buneva, V.N.; Nevinsky, G.A. Mechanism of supercoiled DNA cleavage by human DNA topoisomerase I: Effect of ligand structure on the catalytic stage of the reaction”, *Mol. Biol. (Moscow)* **2003**. *37,* 325–339.

Supplementary Table S19

A rough estimate of the relative contribution of various factors (in terms of affinity, K_d values). characterizing the specific and unspecific interactions of mouse and human topoisomerases with DNA.

Topo	Ligands and conditions		K_d, M	Increasing affinity factor
Mouse	Unspecific	ss	8.5×10^{-5}	
		ds	1.0×10^{-5}	8.5
	Specific	ss	2.0×10^{-6}	50
		ds	7.0×10^{-8}	29
	Strengthening contacts after pre-incubation	ds	1.0×10^{-8}	7
	"Topimage" os supercoiled DNA	ds	10^{-10}	100
Human	Unspecific	ss	5.0×10^{-4}	
		ds	$>1.0\times10^{-3}$	<0.5
	Specific	ss	1.6×10^{-6}	312
		ds	7.0×10^{-8}	23
	Strengthening contacts after pre-incubation	ds	1.0×10^{-8}	7
	"Topimage" os supercoiled DNA	ds	10^{-10}	100

[*]Standard error in experimentally determined K_d values was 10–30%; mean of 3 measurements are given.

References:

1. Bugreev, D.V.; Buneva, V.N.; Nevinsky, G.A. Mechanism of supercoiled DNA cleavage by human DNA topoisomerase I: Effect of ligand structure on the catalytic stage of the reaction", *Mol. Biol. (Moscow)* **2003**. *37*, 325–339.

Supplementary Table S20

Affinity of the first LF DNA-binding site for minimal ligands and their structural components

Ligand	K_d, mM*	ΔG°, kcal/mol***
**Pi**	**5.0±0.5**	**3.2±0.3**
**dRp**	**3.0±0.2**	**3.5±0.2**
**Deoxyribose****	**600.0±60.0**	**0.3±0.03**
**dGMP**	**1.0±0.1·**	**4.2±0.4**
**G-base****	**333.3±30.0**	**7.0±0.07**
**dAMP**	**1.1±0.07**	**4.1±0.3**
**A-base****	**366.7±30.0**	**0.6±0.05**
**TMP**	**1.6±0.1**	**3.9±0.24**
**T-base****	**533.5±50.0**	**0.4±0.04**
**dCMP**	**0.56±0.06**	**4.5±0.5**
**C-base****	**190.0±20.0**	**1.0±0.1**

***Mean±S.E. of three measurements are given**

*******Ortho***phosphate (P_i) and deoxyribophosphate (dRp) or (dRp) and different dNMPs**

*******$\Delta G^o = -RT \times \ln K_d$

References:

1. Guschina, T.A.; Soboleva, S.E.; Nevinsky, G.A. Recognition of specific and nonspecific DNA by human lactoferrin. *J. Mol. Recognit.* **2013**, **26**, 136-148.

Supplementary Table S21
Affinity of the first LF DNA-binding site for single- and double-stranded homo-ODNs and mixed-sequence ODNs

Ligand*	K_d, M**	Ligand	K_d, M	Ligand	K_d, M
d(pA)$_2$	3.7×10^{-4}	d(pT)$_2$	5.0×10^{-4}	d(pC)$_2$	2.7×10^{-4}
d(pA)$_4$	1.0×10^{-4}	d(pT)$_4$	1.7×10^{-4}	d(pC)$_4$	6.5×10^{-5}
d(pA)$_6$	2.0×10^{-5}	d(pT)$_6$	3.3×10^{-5}	d(pC)$_6$	1.1×10^{-5}
d(pA)$_8$	5.0×10^{-6}	d(pT)$_8$	7.3×10^{-6}	d(pC)$_8$	3.0×10^{-6}
d(pA)$_9$	3.3×10^{-6}	–	–	d(pC)$_9$	1.0×10^{-6}
d(pA)$_{10}$	$6.6\times10^{-7*}$	d(pT)$_{10}$	8.3×10^{-7}	d(pC)$_{10}$	4.2×10^{-7}
d(pA)$_{12}$	6.3×10^{-7}	d(pT)$_{12}$	5.8×10^{-7}	d(pC)$_{12}$	3.2×10^{-7}
d(pA)$_{13}$	1.3×10^{-6}	d(pT)$_{13}$	1.0×10^{-6}	d(pC)$_{12}$	3.0×10^{-7}
d(pA)$_{14}$	7.9×10^{-7}	d(pT)$_{14}$	8.3×10^{-7}	d(pC)$_{14}$	2.8×10^{-7}
d(pA)$_{16}$	8.1×10^{-7}	d(pT)$_{16}$	5.7×10^{-7}	d(pC)$_{16}$	2.8×10^{-7}
d(pA)$_{20}$	8.0×10^{-7}	d(pT)$_{20}$	6.0×10^{-7}	d(pC)$_{20}$	2.7×10^{-7}
d[p(Et)T]$_{10}$♣	9.0×10^{-6}	d(pT(pR)$_5$)♣	3.9×10^{-5}	d(pT(pR)$_9$)♣	1.8×10^{-6}
ODN1	8.0×10^{-8}	ODN2	2.2×10^{-7}	ODN3	8.0×10^{-7}
ODN4	3.5×10^{-7}	ODN5	6.4×10^{-7}	ODN6	6.6×10^{-7}
ODN7	6.8×10^{-7}	ODN8	4.5×10^{-7}	-	-
r(pA)$_{10}$	2.3×10^{-7}	r(pU)$_{10}$	1.1×10^{-7}	r(pC)$_{10}$	1.5×10^{-7}

Ligand	K_i, M	Ligand	K_i, M
d(pA)$_4$×d(pT)$_4$	3.2×10^{-5}	d(pA)$_{12}$×d(pT)$_{12}$	9.0×10^{-8}
d(pA)$_6$×d(pT)$_6$	8.7×10^{-6}	d(pA)$_{14}$×d(pT)$_{14}$	3.3×10^{-8}
d(pA)$_8$×d(pT)$_8$	2.0×10^{-6}	d(pA)$_{16}$×d(pT)$_{16}$	3.3×10^{-8}
d(pA)$_{10}$×d(pT)$_{10}$	2.7×10^{-7}	d(pA)$_{20}$×d(pT)$_{20}$	3.0×10^{-8}
ds ODN1**	1.0×10^{-8}	ds ODN2	1.8×10^{-7}
ds ODN3	2.5×10^{-7}	ds ODN4	1.5×10^{-7}
ODN5	3.0×10^{-7}	ODN6	2.8×10^{-7}
ODN7	2.4×10^{-7}	ODN8	2.0×10^{-7}

*Mean of three independent experiments; the error did not exceed 10-15%.

♣R is a tetrahydrofuran analog of abasic deoxyribose; Et, ethyl.

****ODN1, d(pTpApGpApApGpApTpCpApApA); ODN2, d(pApCpTpApCpApGpTpCpTpApCpA); ODN3, d(pCpTpTpTpTpCpCpGpCpCpCpTpC); ODN4, d(pCpTpTpTpTpCpCpGpCpC); ODN5, d(pApTpApTpTpTpApApApG); ODN6, d(pTpGpTpApApGpGpTpTpT); ODN7, d(pGpGpTpTpTpApApTpTpA); ODN8, (pGpTpApTpGpTpTpTpApApApTpApApApTpGpT).**

References:

1. Guschina, T.A.; Soboleva, S.E.; Nevinsky, G.A. Recognition of specific and nonspecific DNA by human lactoferrin. *J. Mol. Recognit.* **2013**, **26**, 136-148.

Supplementary Table S22

Affinity of the first and second HSA DNA-binding site for *ortho*phosphate, dNMPs, single- and double-stranded ODNs

Ligand	*n*	*K*d(1), M*	*K*d(2), M
***Ortho*phosphate**	**0**	**3.0×10^{-3}**	**2.0×10^{-2}-**
**d(pA)**	**1**	**5.6×10^{-6}**	**6.3×10^{-5}**
**d(pA)$_{2}$**	**2**	**8.5×10^{-7}**	**6.5×10^{-6}**
**d(pA)$_{3}$**	**3**	**2.5×10^{-7}**	**2.5×10^{-6}**
**d(pA)$_{4}$**	**4**	**9.7×10^{-8}**	**9.5×10^{-7}**
**d(pA)$_{5}$**	**5**	**9.0×10^{-8}**	**4.9×10^{-7}**
**d(pA)$_{6}$**	**6**	**7.0×10^{-8}**	**7.4 × 10-7**
**d(pA)$_{7}$**	**7**	**5.0×10^{-8}**	**6.0×10^{-7}**
**d(pA)$_{10}$**	**10**	**7.2×10^{-8}**	**4.5×10^{-7}**
**d(pA)$_{11}$**	**11**	**2.8×10^{-8}**	**4.0 × 10-7**
**d(pA)$_{13}$**	**13**	**2.8×10^{-8}**	**3.3×10^{-7}**
**d(pA)$_{24}$**	**24**	**2.4×10^{-8}**	**2.4×10^{-7}**
**d(pT)**	**1**	**1.7×10^{-5}**	**1.0×10^{-3}**
**d(pT)$_{2}$**	**2**	**1.0×10^{-6}**	**3.0×10^{-5}**
**d(pT)$_{3}$**	**3**	**1.8×10^{-8}**	**1.0×10^{-6}**
**d(pT)$_{4}$**	**4**	**7.2×10^{-9}**	**4.8×10^{-7}**
**d(pT)$_{5}$**	**5**	**2.0×10^{-9}**	**2.4 ×10-7**
**d(pT)$_{6}$**	**6**	**1.7×10^{-9}**	**1.8×10^{-7}**
**d(pT)$_{11}$**	**11**	**1.8×10^{-9}**	**1.8×10^{-7}**
**d(pT)$_{12}$**	**12**	**1.7×10^{-9}**	**1.5×10^{-7}**
**d(pT)$_{16}$**	**16**	**1.4×10^{-9}**	**1.0×10^{-7}**
**d(pT)$_{24}$**	**24**	**1.4×10^{-9}**	**8.0×10^{-8}**
**d(pT)$_{30}$**	**30**	**1.6×10^{-9}**	**8.0×10^{-8}**
**d(pC)**	**1**	**4.0×10^{-4}**	**1.8×10^{-2}**
**d(pC)$_{2}$**	**2**	**9.5×10^{-5}**	**3.0×10^{-3}**
**d(pC)$_{3}$**	**3**	**9.5×10^{-6}**	**3.0×10^{-5}**
**d(pC)$_{4}$**	**4**	**6.3×10^{-7}**	**2.0×10^{-6}**
**d(pC)$_{5}$**	**5**	**2.5×10^{-7}**	**1.0×10^{-6}**
**d(pC)$_{6}$**	**6**	**2.4×10^{-7}**	**8.0×10^{-7}**
**d(pC)$_{8}$**	**8**	**1.8×10^{-7}**	**4.0×10^{-7}**
**d(pC)$_{9}$**	**9**	**1.5×10^{-7}**	**2.4×10^{-7}**
**d(pC)$_{10}$**	**10**	**1.5×10^{-7}**	**2.4×10^{-7}**
**d(pC)$_{12}$**	**12**	**1.5×10^{-7}**	**1.8×10^{-7}**
**d(pC)$_{30}$**	**30**	**1.5×10^{-7}**	**1.8×10^{-7}**
**d(pA)$_{2}$×d(pT)$_{2}$**	**2**	**8.1×10^{-7}**	**1.0×10^{-5}**
**d(pA)$_{4}$×d(pT)$_{4}$**	**4**	**1.4×10^{-7}**	**2.4×10^{-6}**
**d(pA)$_{8}$×d(pT)$_{8}$**	**8**	**3.0×10^{-7}**	**3.0×10^{-6}**
**d(pA)$_{12}$×d(pT)$_{12}$**	**12**	**4.0×10^{-7}**	**4.0×10^{-6}**
**d(pA)$_{16}$×d(pT)$_{16}$**	**16**	**5.0×10^{-7}**	**4.5×10^{-6}**
**d(pA)$_{20}$×d(pT)$_{20}$**	**20**	**5.0×10^{-7}**	**4.5×10^{-6}**

Mean of three independent experiments; the error did not exceed 10-15%.

References:

1. Alinovskaya, L.I.; Sedykh, S.E.; Ivanisenko, N.V.; Soboleva, S.E.; Nevinsky, G.A. How human serum albumin recognizes DNA and RNA. *Biol. Chem.* **2018**, *399*, 347-360.

Supplementary Table S23
Thermodynamic parameters (ΔG^o, kcal/mol) characterizing interaction of every of eight mononucleotide units of different oligonucleotides with the fist and the second DNA-binding sites of human serum albumin

Oligo-nucleotide	Gibbs free energy, kcal/mol*								
	Number (*n*) of monomers in $d(pN)_n$ and sum of ΔG^o								
	The first DNA-binding site								
	1	2	3	4	5	6	7	8	Sum
$d(pA)_n$	7.16	1.12	0.7	0.64	0.19	0.07	~0.0	~0.0	9.88
$d(pT)_n$	6.1	1.68	2.38	0.54	0.48	0.10	~0.0	~0.0	11.28
$d(pC)_n$	4.63	0.85	1.36	1.6	0.55	0.013	~0.0	~0.0	9.00
$r(pA)_n$	6.16	2.78	0.87	0.11	0.055	0.025	~0.0	~0.0	10.0
$r(pU)_n$	7.0	0.95	0.81	0.32	0.47	0.27	0.13	~0.0	9.95
$r(pC)_n$	7.24	0.94	1.05	0.50	0.83	0.35	0.25	0.25	11.41
	The second DNA-binding site								
$d(pA)_n$	5.73	1.34	0.57	0.39	0.19	0.15	0.12	~0.0	8.49
$d(pT)_n$	4.1	2.1	2.0	0.43	0.41	0.17	0.15	~0.0	9.36
$d(pC)_n$	1.75	1.10	2.73	1.6	0.41	0.13	0.17	~0.0	7.89
$r(pA)_n$	4.91	1.63	0.97	0.56	0.54	0.22	0.11	0.013	8.95
$r(pU)_n$	5.71	0.23	0.62	0.94	0.20	0.51	0.24	~0.0	8.45
$r(pC)_n$	6.62	0.74	0.26	0.57	0.54	0.17	0.20	0.24	9.34

***Mean of three independent experiments; the error did not exceed 10-15%.**

1. Alinovskaya, L.I.; Sedykh, S.E.; Ivanisenko, N.V.; Soboleva, S.E.; Nevinsky, G.A. How human serum albumin recognizes DNA and RNA. *Biol. Chem.* **2018**, *399*, 347-360.

Supplementary Table S24
The affinity of α-lactalbumin for *ortho*phosphate, dNMP, single- and double-stranded ODNs

Ligand	*n*	K_d. M*	Ligand	*n*	K_d. M*
***Ortho*phosthate**	**0**	**1.0 × 10^{-3}**	**-**	**-**	**-**
**d(pA)**	**1**	**5.0 × 10^{-5}**	**d(pT)**	**1**	**3.0 × 10^{-4}**
**$d(pA)_2$**	**2**	**5.3 × 10^{-6}**	**$d(pT)_2$**	**2**	**6.5 × 10^{-5}**
**$d(pA)_3$**	**3**	**2.8 × 10^{-6}**	**$d(pT)_3$**	**3**	**9.4 × 10^{-6}**
**$d(pA)_4$**	**4**	**6.6 × 10^{-7}**	**$d(pT)_4$**	**4**	**1.8 × 10^{-6}**
**$d(pA)_5$**	**5**	**2.4 × 10^{-7}**	**$d(pT)_5$**	**5**	**1.4 × 10^{-6}**
**$d(pA)_6$**	**6**	**8.8 × 10^{-8}**	**$d(pT)_6$**	**6**	**9.0 × 10^{-7}**
**$d(pA)_7$**	**7**	**8.8 × 10-8**	**$d(pT)_8$**	**8**	**1.0 10^{-6}**
**$d(pA)_8$**	**8**	**1.0 × 10^{-7}**	**$d(pT)_{10}$**	**10**	**2.4 × 10^{-6}**
**$d(pA)_{11}$**	**11**	**1.3 × 10^{-7}**	**$d(pT)_{11}$**	**11**	**1.1 × 10^{-5}**
**$d(pA)_{13}$**	**13**	**1.6 × 10^{-7}**	**$d(pT)_{12}$**	**12**	**1.5 × 10^{-5}**
**$d(pA)_{16}$**	**16**	**1.2 × 10^{-6}**	**$d(pT)_{14}$**	**14**	**1.9 × 10^{-5}**
**$d(pA)_{20}$**	**20**	**2.2 × 10^{-6}**	**$d(pT)_{16}$**	**16**	**8.6 × 10^{-5}**
**$d(pA)_{24}$**	**24**	**2.9 × 10^{-6}**	**$d(pT)_{24}$**	**24**	**1.1 × 10^{-4}**
**d(pC)**	**1**	**1.2 × 10^{-4}**	**$d(pC)_8$**	**8**	**5.0 × 10^{-7}**
**$d(pC)_2$**	**2**	**3.0 × 10^{-5}**	**$d(pC)_9$**	**9**	**5.3 × 10^{-7}**
**$d(pC)_3$**	**3**	**1.2 × 10^{-6}**	**$d(pC)_{10}$**	**10**	**2.6 × 10^{-6}**
**$d(pC)_4$**	**4**	**5.5 × 10^{-7}**	**$d(pC)_{12}$**	**12**	**1.2 × 10^{-5}**
**$d(pC)_5$**	**5**	**2.2 × 10^{-7}**	**$d(pC)_{16}$**	**16**	**1.6 × 10^{-4}**
**$d(pC)_6$**	**6**	**2.1 × 10^{-7}**	**$d(pC)_{24}$**	**24**	**1.8 × 10^{-4}**
**$d(pA)_6 \times d(pT)_6$**	**6**	**1.8×10^{-7}**	**$d(pA)_{16} \times d(pT)_{16}$**	**16**	**8.5×10^{-6}**
**$d(pA)_{12} \times d(pT)_{12}$**	**12**	**1.2×10^{-6}**	**$d(pA)_{20} \times d(pT)_{20}$**	**20**	**1.7×10^{-5}**

*** The average of three independent experiments: the determination error did not exceed 10-15%.**

References:

1. Nevinsky, G.A.; Alinovskaya, L.I.; Sedykh, S.E.; Soboleva, S.E.; How human alpha-lactalbumin recognize DNA and RNA. *Biochem. Anal. Biochem.* **2018**, *7*, 4.

Supplementary Table S25

$K_d(1)$ and $K_d(2)$ values, characterizing affinity of different single stranded homo-ODNs to IgGs from the blood sera of patients with multiple sclerosis

Ligand	$K_d(1)$, M	$K_d(2)$, M	Ligand	$K_d(1)$, M	$K_d(2)$, M	Ligand	$K_d(1)$, M	$K_d(2)$, M
Ortho-phosphate	1.0×10^{-3}	3.3×10^{-3}	d(pR)**	1.4×10^{-4}	4.3×10^{-4}	-	-	-
dAMP	6.3×10^{-7}	1.7×10^{-4}	dCMP	3.8×10^{-6}	2.8×10^{-4}	dTMP	1.9×10^{-6}	1.2×10^{-5}
$d(pA)_2$	1.5×10^{-7}	1.4×10^{-6}	$d(pC)_2$	3.1×10^{-7}	2.3×10^{-6}	$d(pT)_2$	7.5×10^{-7}	1.4×10^{-6}
$d(pA)_4$	5.8×10^{-8}	1.0×10^{-6}	$d(pC)_3$	1.8×10^{-7}	1.7×10^{-6}	$d(pT)_3$	1.5×10^{-8}	8.1×10^{-7}
$d(pA)_6$	2.2×10^{-8}	7.2×10^{-7}	$d(pC)_4$	5.7×10^{-8}	9.0×10^{-7}	$d(pT)_4$	1.6×10^{-8}	6.0×10^{-7}
$d(pA)_8$	2.2×10^{-8}	6.7×10^{-7}	$d(pC)_6$	1.0×10^{-8}	7.9×10^{-7}	$d(pT)_6$	1.0×10^{-9}	2.7×10^{-7}
$d(pA)_9$	2.2×10^{-8}	4.5×10^{-7}	$d(pC)_8$	3.7×10^{-9}	6.6×10^{-7}	$d(pT)_8$	1.1×10^{-8}	2.3×10^{-7}
$d(pA)_{10}$	2.1×10^{-8}				4.6×10^{-7}	$d(pT)_{10}$	1.4×10^{-8}	2.4×10^{-7}
$d(pA)_{12}$	1.9×10^{-8}	3.4×10^{-7}	$d(pC)_{10}$	3.2×10^{-9}	3.9×10^{-7}	$d(pT)_{11}$	1.4×10^{-8}	2.9×10^{-7}
$d(pA)_{13}$	1.9×10^{-8}	3.1×10^{-7}	$d(pC)_{12}$	3.1×10^{-9}	3.8×10^{-7}	$d(pT)_{16}$	1.3×10^{-8}	$2{\cdot}.0\times10^{-7}$
$d(pA)_{15}$	1.9×10^{-8}	2.9×10^{-7}	$d(pC)_{14}$	2.9×10^{-9}	3.6×10^{-7}	$d(pT)_{24}$	1.3×10^{-8}	1.6×10^{-7}
$d(pA)_{16}$	1.9×10^{-8}	2.3×10^{-7}	$d(pC)_{16}$	3.0×10^{-9}	3.4×10^{-7}	-	-	-
$d(pA)_{20}$	2.0×10^{-8}	2.0×10^{-7}	$d(pTHF)_6$ ϒ	3.9×10^{-7}	1.0×10^{-4}	$d[T(pTHF)_5]$	5.1×10^{-9}	1.5×10^{-6}

***Mean ± S.E. of two independent measurements are given; the error did not exceed 10-215%.**
****Deoxyribosephosphate, R is deoxyribose**
ϒTHF is a tetrahydrofuran

References:

1. Andreev, S.K.; Buneva V.N.; Nevinsky, G.A. How human IgGs against DNA recognize oligonucleotides and DNA. *J. Mol. Recognit.* **2016**, *29*, 596-610.

Supplementary Table S26

K_d(1) and K_d(2) values, characterizing affinity of different single stranded hetero-ODNs to IgG from the blood sera of patients with multiple sclerosis *.

Ligand	Number of units (*n*)	$K_d(1)$, M	$K_d(2)$, M
d(pTC)	2	8.2×10^{-7}	3.8×10^{-6}
d(pTGC)	3	1.0×10^{-7}	2.1×10^{-6}
d(pTTGC)	4	2.2×10^{-7}	5.2×10^{-6}
d(pTTGG)	4	8.0×10^{-8}	2.4×10^{-6}
d(pGAATTC)	6	5.3×10^{-8}	2.5×10^{-6}
d(pAAAGCAGG)	8	2.3×10^{-8}	2.2×10^{-6}
d(pTTCTTCTTC)	9	1.3×10^{-8}	1.3×10^{-6}
d(pTGATGATGA)	9	2.8×10^{-8}	2.9×10^{-6}
d(pGATGTCTGGTCCA)	13	4.5×10^{-9}	5.2×10^{-7}

***Mean ± S.E. of two independent measurements are given; the error did not exceed 10-15%.**

References:

1. Andreev, S.K.; Buneva V.N.; Nevinsky, G.A. How human IgGs against DNA recognize oligonucleotides and DNA. *J. Mol. Recognit.* **2016**, *29*, 596-610.
